# Effects of External Oiling and Rehabilitation on Hematological, Biochemical, and Blood Gas Analytes in Ring-Billed Gulls (*Larus delawarensis*)

**DOI:** 10.3389/fvets.2019.00405

**Published:** 2019-11-19

**Authors:** Nicholas G. Dannemiller, Katherine E. Horak, Jeremy W. Ellis, Nicole L. Barrett, Lisa L. Wolfe, Susan A. Shriner

**Affiliations:** ^1^US Department of Agriculture, Animal Plant Health Inspection Service, Wildlife Services, National Wildlife Research Center, Fort Collins, CO, United States; ^2^Department of Clinical Sciences, Colorado State University, Fort Collins, CO, United States; ^3^Wildlife Health Program, Colorado Parks and Wildlife, Fort Collins, CO, United States

**Keywords:** oil toxicity, wildlife rehabilitation, hemolytic anemia, immune suppression, blood gas, Ring-billed Gull

## Abstract

Avian species experience extensive morbidity and mortality following large-scale oil spills, often resulting in oiled birds being rescued, and admitted to rehabilitation. Our objective was to experimentally establish time-specific, descriptive blood analyte data following sublethal oil exposure and subsequent rehabilitation. Thirty wild Ring-billed Gulls (*Larus delawarensis*) were randomly allocated to three treatment groups of 10 birds each. One treatment group served as controls and two treatment groups were externally oiled daily for 3 days with weathered MC252 oil collected from the Deepwater Horizon oil spill, mimicking the upper threshold of the US Fish and Wildlife Service's moderate oiling classification. Following external oiling, one oiled treatment group was cleaned via standard rehabilitation practices. Serial venous blood samples were collected for a month to measure packed cell volume, total solids, blood gas and select plasma biochemistry analytes, total white blood cell estimates and differentials, and reticulocyte estimates. We found that both sublethal oil exposure and aspects of captivity were associated with a mild non-regenerative anemia. No other differences in venous blood gas and biochemical analytes as well as white blood cell concentrations were observed among the three groups. These findings suggest that the mild anemia seen in oiled birds undergoing rehabilitation is possibly multifactorial and that moderately oiled gulls have subtle, but potentially not insignificant clinicopathological abnormalities following sublethal oil exposure. Oiled gulls did not develop any clinicopathological derangements post-rehabilitation, suggesting current standard practices for rehabilitation cause minimal morbidity in clinically stable, moderately oiled gulls.

## Introduction

The negative impact of oil spills on wildlife, particularly birds, has been widely recognized. Aquatic and semi-aquatic avian populations have experienced extensive morbidity and mortality following many large-scale oil spills, such as the Exxon Valdez (1), Prestige (2), and most recently, Deepwater Horizon (3, 4). However, crude oil's true impact on birds is likely larger than the reported acute mortality estimates (5, 6). Chronic oil exposure has been implicated in avian population declines long after the initial mass mortality following an oil spill, slowing population recovery despite clean up and environmental restoration (7). Thus, a stronger understanding of the effects of sublethal external oil exposure (i.e., external oiling that does not lead to acute death) on individual birds is warranted to better characterize avian oil toxicosis as well as the broader impacts of oil spills.

Sublethal oil exposure on birds causes a wide range of adverse effects including: hemolytic anemia; decreased nutrient absorption; altered physiological stress responses; renal, hematopoietic, and hepatic damage; and immunosuppression (8–12). Of these adverse effects, hemolytic anemia has been most frequently demonstrated in birds exposed to crude oil. Hemolytic anemia secondary to oil exposure is likely caused by oxidative damage mediated by metabolites of polycyclic aromatic hydrocarbons generated from cytochrome P450 enzymes in the livers of oiled birds (13, 14). Oil induced hemolytic anemia is characterized by red blood cell (RBC) regeneration, increased numbers of reticulocytes, and the development of Heinz bodies from oxidative damage to hemoglobin; however, the kinetics of these abnormalities is not well-characterized. Although the adverse effect of oil exposure on RBCs has been well-investigated, there are limited reports on the effect of oil on white blood cells (WBC) (15, 16). Previous studies have shown birds exposed to oil often exhibit reduction in total WBC counts and lymphocytes suggestive of immunosuppression as well as markedly elevated heterophil counts and heterophil/lymphocyte ratios (15, 16). There are currently no reports on the effect of oil exposure on the blood gas and acid-base status of birds.

Due to the development of protocol-driven response and recovery efforts, oiled birds are increasingly rescued and admitted to rehabilitation following oil spills (17). While on-going research continues to target aspects of emergency medicine and critical care for improvement, rehabilitation alone is an inherently stressful sequence of events for oiled birds. To date, no studies have evaluated the effect of current rehabilitation procedures on oiled birds. Knowledge of the clinicopathological abnormalities observed in birds following prolonged sublethal oil exposure, the effect rehabilitation has on these abnormalities, and the overall chronology of these abnormalities would be invaluable to veterinarians treating and managing oiled birds. However, given the temporal and spatial unpredictability of oil spills, this knowledge is difficult to acquire from wild birds *in situ*. While most experimental research on oil exposure has traditionally focused on oral dosing, less work has been conducted on sublethal external oiling. A recent suite of papers assessing avian exposure to Deepwater Horizon crude oil (18) has shown myriad impacts from sublethal external oiling, including flight impairment, reduced weight gain, altered metabolome, oxidative damage, changes in plasma biochemistry, changes in organ weights and histopathology, and cardiac dysfunction (19–24). Consequently, further exploration of the impacts of external oiling is warranted.

The objective of our study was to establish time-specific estimates of hematological, biochemical, and venous blood gas analytes to not only improve the point-of-care assessment of oiled birds, but also help better understand the pathogenesis of oil toxicosis from external oiling. Under experimental conditions and through serial blood sampling, our study characterizes post-exposure and post-rehabilitation changes in select blood analytes for birds externally oiled with weathered crude oil from the Deepwater Horizon oil spill. We also establish reference intervals for wild Ring-billed Gulls (*Larus delawarensis*), a common species found in coastal areas at risk of oil spills. We hypothesized that sublethal oil exposure would cause anemia and immunosuppression in externally oiled birds and that rehabilitated birds would return to pre-exposure values more quickly than post-exposure, non-rehabilitated birds.

## Methods

### Gull Capture and Husbandry

All aspects of this study, including bird capture, transport, quarantine, husbandry, oiling, and sampling, were performed according to procedures approved by the Institutional Animal Care and Use Committee of the US Department of Agriculture, Animal and Plant Health Inspection Service, National Wildlife Research Center (NWRC; Approval 2806). Thirty-five Ring-billed Gulls (RBGU) were captured in Larimer County, CO using air cannon nets and net guns and transported to the NWRC, Fort Collins, CO in large ventilated crates. Birds were not captured in extreme weather conditions and transport time was <1 h. Capture was conducted under the authority of US Fish and Wildlife Service (USFWS) Migratory Bird Permit #MB019065 as well as Colorado Parks and Wildlife scientific collection license #18TRb2433.

Upon arrival at the NWRC, captured RBGUs were given a complete and thorough physical exam by the NWRC attending veterinarian, weighed, individually marked with a unique leg band, and a baseline blood sample was collected. Body temperature was also measured using a VetOne digital cloacal thermometer (MWI Animal Health, Aurora, CO). Given RBGUs are not sexually dimorphic, the sex distribution of the captured birds was unknown. Most birds were characterized as breeding adults based on plumage, but a few had markings that were consistent with either non-breeding adult or second winter plumage. Throughout the study, birds were group housed in large, netted outdoor flight pens (18.29 × 37.80 m) that contained artificial pools, heat lamps, as well as platforms and other objects that allowed for natural perching and loafing. During testing, RBGUs were offered a maintenance diet of commercial avian cubes (Mazuri Fish Analog 50/10 Frozen 5T8, St. Louis, MO), raw golden shiner minnows (*Notemigonus crysoleucas*, I.F. Anderson Farms, Inc., Lonoke, AR), and canned mackerel *ad libutum* two times per day. At the conclusion of this study, all surviving birds were transferred as study animals to other research projects or released near the capture site in Larimer County, CO.

### Oil Source

MC252 oil collected during the Deepwater Horizon oil spill and artificially weathered (i.e., weathered in the laboratory prior to receipt for use in studies) was used to moderately externally oil two of the three treatments groups. Oil was artificially weathered by TDI-Brooks International, Inc. and B&B Laboratories (College Station, TX) and shipped to NWRC by Ecochem, Inc. (Tukwila, WA) under chain of custody.

### External Oiling and Rehabilitation

After acclimation, 30 RBGUs were randomly allocated to three treatment groups of 10 birds each. *A priori* power analyses determined 10 birds per treatment group would adequately minimize a Type II statistical error (power = 0.996, given effect size = 0.8 and α = 0.05). Two treatment groups were externally oiled daily for 3 days with 5–7 mL of oil applied per day with a paintbrush to the breast, wing edges, and tail feathers mimicking the upper threshold of the USFWS moderate oiling classification for birds. This level of oiling is generally described as 14–40% surface area coverage. In general, oil was applied to body areas that would be exposed to an oil sheen for surface feeding birds such as RBGUs. After 3 days, oiled RBGUs received a cumulative total of 15–21 mL of oil. The third treatment group served as controls and were handled in the same manner as the other two groups but were painted with water instead of oil for 3 days. Nine days after initial oiling, one of the oiled treatment groups was cleaned following standard practices for rehabilitating oiled birds over a 48-h period. Briefly, oiled birds were washed in sequential tubs filled with diluted detergent (1-2%) in softened water (2–3 grains of hardness) heated to 106-110°F (41–43°C) until the water remained clear. Birds were then rinsed with softened water at a temperature of 106-112°F and allowed to rest in a clean and well-ventilated brooder box lined with warm towels and equipped with water for drinking while being dried with heat lamps. During the drying process, air temperature was maintained between 90 and 95°F (32-35°C), with the birds being carefully monitored for signs of hypothermia or hyperthermia. The second oiled treatment group remained externally oiled until the conclusion of the study and then was cleaned using the same methods as previously described.

### Serial Venous Blood Sampling

In order to characterize changes over time, blood samples (200–300 μL), weights, and temperatures were collected from RBGUs immediately prior to the external oiling and then 7, 15, 22, and 31 days post-oiling (DPO). Venous blood was anaerobically and aseptically collected using a 1.0 inch, 22-gauge needle, and heparinized syringes from either the left or right brachial, distal metatarsal, or external jugular veins. Blood samples were immediately divided into two subsamples. The first subsample was stored in microtainers to later measure packed cell volume (PCV) and total solids using a PCV chart and refractometer as well as visually assess for presence of plasma hemolysis, following high-speed centrifugation of blood-filled microhematocrit tubes. The second subsample was used for instant venous blood gas and select plasma biochemistry analysis, using the Element POC Blood Gas & Electrolyte Analyzer (EPOC; Heska Corporation, Loveland, CO), a handheld veterinary point-of-care analyzer.

Venous blood gas and plasma biochemistry analytes of interest included: hematocrit; plasma concentrations of sodium, potassium, chloride, ionized calcium, lactate, glucose, and bicarbonate; pH; and venous pO_2_ and pCO_2_. Because the EPOC performed all analyses at 37°C, pH, pCO_2_, pO_2_, were temperature corrected (TC) to reflect the RBGU's respective body temperatures via the following equations:

$$\begin{array}{l} TC\;pH=pH+\left(0.0147*\Delta T\right)\\ TC\;pCO_{2}=pCO_{2}*10^{\left(-0.019*\Delta T\right)}\\ TC\;pO_{2}=\;pO_{2}*10^{\left(-0.0058*\Delta T\right)} \end{array}$$

where ΔT = 37°C−body temperature (25). The TC bicarbonate concentration was calculated via the Henderson-Hesselbach equation using TC pH and pCO_2_ values as well as the solubility coefficient for CO_2_ and pK (logarithmic acid dissociation constant) (25). Due to the effects of pH on ionized calcium, the following correction was applied (26):

$$\begin{array}{l} TC\;Ionized\;Calcium\\ =Ionized\;Calcium*\left(1+\left(0.53\left(pH-TC\;pH\right)\right)\right) \end{array}$$

Anion gap was calculated via the following equation:

$$\begin{array}{l} Anion\;Gap=\left(Sodium+Potassium\right)\\ -\left(Chloride+TC\;Bicarbonate\right) \end{array}$$

Two duplicate blood smears were also made each time blood was drawn and subsequently submitted to the Colorado State University Veterinary Diagnostic Lab (Fort Collins, Colorado) for processing. Total WBC estimates were derived using an internal standard operating procedure for avian WBC estimates due to the unavailability of a whole blood sample. Briefly, all WBCs seen in ten 50× oil fields were counted and then divided by 10 to obtain the average WBC count per 50× oil field. This average was then used in the following equation:

$$\begin{array}{l} \text{Total WBC}/\mu \text{l}=\;5,000\text{ WBC}\\ +\;3,000\text{ WBC x }\left(\text{Average WBC}/50\times \text{ oil field }-\;1\right) \end{array}$$

This WBC estimate formula was developed from internal unpublished studies at the Colorado State University Veterinary Diagnostic Laboratory and has not been validated or verified. WBC differential was performed by calculating the percentage of each leukocyte type seen in a sample of 100 leukocytes under 1,000× oil immersion (27). Reticulocyte estimates were generated by enumerating aggregate reticulocytes and ring forms using previously described methods (28).

### Statistical Analysis

Physical, hematological, biochemical, and blood gas data collected from all 30 RBGUs immediately prior to the external oiling were aggregated to generate reference intervals. Given our small sample size and that some analytes had non-parametric distributions, robust estimation of the 90% confidence interval of reference limits was employed as suggested by Friedrichs et al. (29). Robust estimation of the 90% confidence interval was done using a bias corrected bootstrap method based on 10,000 bootstrap replicates. Median, minimum, and maximum values were also calculated and reported to allow informed clinical decision-making (29). For subsequent DPO, mean, median, standard deviation, minimum, and maximum were calculated for each treatment group separately. Data were also analyzed using multiple linear mixed models (LMM) for repeated measures with treatment group, DPO, and the interaction between treatment group and DPO as predictor variables, each of the various clinical analytes as the response variable, and the individual RBGU as a random effect. A separate LMM was run for each clinical analyte with the false discovery rate controlled via the Benjamini–Hochberg procedure (30). Hematocrit as determined by EPOC, hereafter HCT (EPOC), and PCV determined manually, hereafter PCV (spun), were compared using a paired *t*-test, Lin's concordance correlation coefficient (31), and a Bland-Altman plot (32). All statistics were performed in the statistical software program R version 3.5 (33) with the 90% confidence intervals being generated with the “boot” package and LMMs being fitted using the “lme4” package (34).

## Results

Physical, hematological, biochemical, and blood gas analyte values from baseline (0 DPO) sampling showed no obvious clinical abnormalities in the RBGUs (Table 1). Of the 30 RBGUs included in this study, 28 survived through its conclusion: one rehabilitated-oiled RBGU died between 7 and 15 DPO and one non-rehabilitated-oiled RBGU died between 22 and 31 DPO. Necropsies and histopathology of the deceased birds found the cause of death was not due to sublethal external oil exposure. LMMs were not run for monocytes, eosinophils, and basophils given their mean and median were 0 × 10^3^/μL on 7, 15, 22, and 31 DPO.

**Table 1:** Reference intervals and summary statistics for body temperature and select venous blood analytes of wild Ring-billed Gulls (*Larus delawarensis*).

**Analytes**	**Reference interval**	**Median**	**Mean ± SD**	**(Min, Max)**
Body temperature (°C)	42.2–42.5	42.4	42.4 ± 0.5	(41.4, 43.4)
PCV (spun) (%)	51–54	52	53 ± 5	(43, 60)
Hematocrit (EPOC) (%)	42–43	43	43 ± 3	(36, 48)
Total Solids (g/dL)	4.2–4.5	4.1	4.3 ± 0.6	(3.2, 5.8)
Sodium (mmol/L)	156–157	156	156 ± 2	(152, 160)
Potassium (mmol/L)	2.7–2.9	2.8	2.8 ± 0.4	(2.2, 3.7)
Chloride (mmol/L)	120–122	120	121 ± 4	(113, 129)
TC ionized calcium (mmol/L)	1.1–1.2	1.1	1.1 ± 0.1	(0.8, 1.3)
TC anion gap	20–22	22	21 ± 3	(14, 31)
Lactate (mmol/L)	4.4–5.7	4.6	4.9 ± 2.1	(2.4, 12.7)
Glucose (mg/dL)	322–351	334	336 ± 48	(244, 424)
TC pH	7.39–7.43	7.40	7.41 ± 0.06	(7.30, 7.60)
TC pCO_2_ (mmHg)	29–32	30	31 ± 5	(21, 40)
TC pO_2_ (mmHg)	48–52	49	50 ± 6	(39, 62)
TC bicarbonate (mmol/L)	16–18	16	17 ± 3	(9, 23)
Total WBC (×10^3^/μL)	10.0–11.0	11.0	10.6 ± 1.8	(7.0, 13.0)
Heterophils (×10^3^/μL)	4.7–5.3	4.9	5.0 ± 1.1	(3.0, 7.7)
Lymphocytes (×10^3^/μL)	2.3–2.6	2.6	2.5 ± 0.4	(1.7, 3.3)
Monocytes (×10^3^/μL)	0.0–0.1	0.1	0.1 ± 0.1	(0, 0.2)
Eosinophils (×10^3^/μL)	0.0–0.0	0.0	0.0 ± 0.0	(0.0, 0.0)
Basophils (×10^3^/μL)	0.0–0.0	0.0	0.0 ± 0.0	(0.0, 0.0)
Reticulocytes (%)	0.7–1.0	1.0	1.0 ± 0.4	(0.0, 2.0)

*Reference intervals are based on 30 wild caught birds (weight range = 404–550 g, median = 471 g, mean = 466 g) sampled immediately prior to external oiling. Reference intervals are 90% confidence intervals estimated via a bias corrected bootstrap method based on 10,000 bootstrap replicates. TC, temperature corrected; SD, standard deviation*.

PCV (spun) and HCT (EPOC) of both the rehabilitated and non-rehabilitated-oiled groups were significantly lower when compared to the control group (Table 2). On average, non-rehabilitated-oiled RBGUs had 4 ± 0.8% lower PCV (spun) than controls and rehabilitated-oiled RBGUs had 3 ± 0.8% lower PCV (spun) than controls. Similarly, non-rehabilitated-oiled RBGUs had 5 ± 0.8% lower HCT (EPOC) than controls and rehabilitated-oiled RBGUs had 4 ± 0.8% lower HCT (EPOC) than controls on average. PCV (spun) and HCT (EPOC) were also significantly lower over time for all groups (Table 2). All treatment groups had lower PCV (spun) and HCT (EPOC) on 7, 15, 22, and 31 DPO compared to 0 DPO (Table 3). HCT (EPOC), but not PCV (spun), exhibited a significant interaction between non-rehabilitated-oiled group and time (Table 2); when compared to the control group, non-rehabilitated-oiled RBGUs' HCT (EPOC) decreased 0.2% more over time. Obvious plasma hemolysis was not visually observed following high-speed centrifugation of blood-filled microhematocrit tubes for any treatment group.

Table 2Linear mixed model results for clinical analytes of Ring-billed Gulls (*Larus delawarensis*) in three treatment groups (Control gulls, Oiled/Non-Rehabilitated gulls, and Oiled/Rehabilitated gulls).

**Table:** 

		**Treatment Non-rehab**		**SE**		**DF**	***t***	***p*-value**		**Treatment Rehab**	**SE**	**DF**		***t***	***p*-value**
Weight		−37.1		21.1		30.7	−1.8	0.088		3.2	21.1	30.8		0.2	0.882
Body temperature		0.0		0.2		56.9	−0.1	0.928		−0.1	0.2	57.3		−0.6	0.547
PCV (spun)		−4.2		1.1		142.0	−3.7	<0.001*		−3.5	1.1	142.0		−3.1	0.003*
Hematocrit (EPOC)		−5.1		1.1		24.3	−4.5	<0.001*		−4.1	1.1	24.8		−3.6	0.001*
Total solids		0.0		0.0		0.0	−0.3	0.729		0.0	0.0	0.0		−0.7	0.468
Sodium		0.6		0.8		18.6	0.8	0.439		−0.2	0.8	19.0		−0.2	0.827
Potassium		0.0		0.2		114.9	−0.1	0.900		0.1	0.2	115.4		0.7	0.508
Chloride		0.4		1.2		74.2	0.4	0.711		1.1	1.2	74.8		0.9	0.364
TC ionized calcium		0.0		0.0		98.5	0.9	0.382		0.0	0.0	99.2		−1.2	0.239
TC anion gap		−1.0		1.0		115.9	−1.0	0.339		−1.7	1.0	116.5		−1.6	0.104
Lactate		0.2		0.7		59.8	0.3	0.746		−0.7	0.7	60.2		−0.9	0.347
Glucose		−27.3		18.5		67.8	−1.5	0.146		−40.7	18.6	68.3		−2.2	0.032
TC pH		0.0		0.0		0.0	−0.4	0.699		0.0	0.0	0.0		−0.1	0.928
TC pCO_2_		0.6		2.0		69.4	0.3	0.800		−0.8	2.0	69.9		−0.4	0.689
TC pO_2_		−0.4		1.7		113.0	−0.2	0.828		1.7	1.7	113.7		1.0	0.310
TC bicarbonate		0.1		0.9		91.1	0.1	0.926		−0.5	0.9	91.7		−0.5	0.600
Total WBC		0.7		0.6		138.0	1.1	0.268		−0.7	0.6	138.0		−1.2	0.252
Heterophils		0.0		0.0		0.0	1.1	0.278		0.0	0.0	0.0		−0.3	0.759
Lymphocytes		0.1		0.2		112.6	0.4	0.691		−0.2	0.2	113.0		−1.5	0.128
Reticulocytes		0.0		0.0		0.0	2.4	0.018*		0.0	0.0	0.0		0.6	0.562

**Table:** 

**Analytes**	**DPO**	**SE**	**DF**	***t***	***p*****-value**	**Non-rehab: DPO**	**SE**	**DF**	***t***	***p*****-value**	**Rehab: DPO**	**SE**	**DF**	***t***	***p*****-value**
Weight	0.0	0.2	113.1	1.8	0.078	0.6	0.4	113.2	1.8	0.078	−0.1	0.4	113.5	−0.3	0.777
Body temperature	0.0	0.0	110.6	−0.4	0.686	0.0	0.0	111.0	1.8	0.069	0.0	0.0	113.0	0.9	0.349
PCV (spun)	−0.1	0.0	142.0	−2.2	0.032*	−0.1	0.1	108.3	−1.1	0.294	0.0	0.1	109.8	−0.2	0.871
Hematocrit (EPOC)	−0.1	0.0	114.5	−3.6	<0.001*	−0.2	0.1	110.5	−2.9	0.005*	0.0	0.1	112.6	−0.3	0.733
Total solids	0.0	0.0	0.0	0.8	0.449	0.0	0.0	0.0	0.2	0.819	0.0	0.0	0.0	−0.1	0.938
Sodium	0.0	0.0	105.8	−1.0	0.315	0.1	0.0	101.8	1.7	0.095	0.1	0.0	104.4	1.8	0.078
Potassium	0.0	0.0	112.1	0.9	0.363	0.0	0.0	113.0	0.4	0.677	0.0	0.0	115.5	0.6	0.551
Chloride	0.0	1.2	74.8	0.9	0.364	0.1	0.1	108.2	1.0	0.306	0.0	0.1	110.9	−0.8	0.399
TC ionized calcium	0.0	0.0	112.5	1.3	0.192	0.0	0.0	113.2	0.4	0.692	0.0	0.0	115.7	1.4	0.158
TC anion gap	0.0	0.0	112.9	−0.6	0.579	0.0	0.1	113.8	0.9	0.377	0.1	0.1	116.2	1.5	0.130
Lactate	0.0	0.0	112.7	0.7	0.483	0.0	0.0	113.1	0.2	0.834	0.0	0.0	115.0	1.2	0.244
Glucose	−0.3	0.6	112.4	−0.6	0.547	0.0	0.8	112.8	0.1	0.955	1.0	0.8	115.0	1.2	0.241
TC pH	0.0	0.0	0.0	−0.6	0.568	0.0	0.0	0.0	−1.8	0.083	0.0	0.0	0.0	−1.6	0.108
TC pCO_2_	0.1	0.1	112.0	1.0	0.314	0.1	0.1	112.5	1.2	0.234	0.2	0.1	114.7	2.4	0.016
TC pO_2_	0.1	0.1	111.0	1.0	0.315	0.0	0.1	112.0	−0.2	0.830	−0.1	0.1	114.5	−1.2	0.235
TC bicarbonate	0.0	0.0	112.7	0.4	0.667	0.0	0.0	113.4	−0.7	0.497	0.0	0.0	115.8	1.0	0.311
Total WBC	0.0	0.0	138.0	0.1	0.931	0.0	0.0	138.0	−1.0	0.301	0.0	0.0	138.0	0.4	0.687
Heterophils	0.0	0.0	0.0	−0.3	0.792	0.0	0.0	0.0	0.0	0.989	0.0	0.0	0.0	1.4	0.168
Lymphocytes	0.0	0.0	112.6	−0.5	0.649	0.0	0.0	114.4	−0.2	0.844	0.0	0.0	114.6	0.7	0.500
Reticulocytes	0.0	0.0	0.0	1.9	0.066	0.0	0.0	0.0	0.0	0.975	0.0	0.0	0.0	−0.7	0.516

Repeated measures of each analyte were modeled with Treatment (Control, Non-Rehab, Rehab), days post-oiling (DPO), and the interaction between Treatment and DPO as predictor variables and individual gulls as a random effect. A separate mixed model was run for each analyte with statistical significance determined via the Benjamini–Hochberg. p-values with an * are statistically significant. TC, temperature corrected; SE, standard error; DF, degrees of freedom.

**Table 3:** Post-oiling summary statistics for Ring-billed Gull (*Larus delawarensis*) weight, body temperature, and select venous blood analytes stratified by treatment group (Control gulls, Oiled/Non-Rehabilitated gulls, and Oiled/Rehabilitated gulls) and days post-oiling (DPO).

		**7 DPO**			**15 DPO**		
**Analytes**	**Group**	**Median**	**Mean ± SD**	**(Min, Max)**	**Median**	**Mean ± SD**	**(Min, Max)**
Weight (g)	Control	435	462 ± 59	(402, 552)	449	487 ± 62	(416, 580)
	Oiled	418	424 ± 41	(372, 484)	425	428 ± 43	(380, 496)
	Rehab	449	447 ± 40	(394, 530)	468	465 ± 46	(400, 456)
Body temperature (°C)	Control	42.3	42.5 ± 0.6	(41.8, 43.8)	42.0	42.5 ± 0.4	(42, 43)
	Oiled	42.2	42.2 ± 0.3	(41.8, 42.7)	43.0	43 ± 0.6	(41.6, 43.7)
	Rehab	42.4	42.3 ± 0.6	(41.3, 43.3)	43.0	43 ± 0.5	(42, 43.5)
PCV (spun) (%)	Control	48	47 ± 2	(44, 51)	48	49 ± 3	(43, 56)
	Oiled	43	42 ± 3	(35, 48)	47	46 ± 5	(32, 51)
	Rehab	43	42 ± 3	(35, 47)	41	42 ± 4	(38, 50)
Hematocrit (EPOC) (%)	Control	41	41 ± 2	(39, 43)	42	41 ± 3	(37, 45)
	Oiled	37	36 ± 3	(29, 39)	38	36 ± 5	(23, 41)
	Rehab	38	37 ± 3	(30, 40)	36	36 ± 3	(32, 40)
Total Solids (g/dL)	Control	4.0	3.9 ± 0.5	(3, 4.8)	4.4	4.5 ± 0.8	(3.4, 6.2)
	Oiled	4.0	3.9 ± 0.5	(3.2, 4.6)	5.3	4.9 ± 0.9	(3.4, 5.9)
	Rehab	3.5	3.7 ± 0.6	(3, 4.8)	4.3	4.4 ± 0.8	(3, 5.6)
Sodium (mmol/L)	Control	156	156 ± 1	(155, 158)	156	155 ± 2	(152, 158)
	Oiled	157	156 ± 1	(154, 158)	158	157 ± 2	(155, 159)
	Rehab	157	155 ± 7	(137, 160)	156	156 ± 2	(154, 160)
Potassium (mmol/L)	Control	3.0	3 ± 0.3	(2.5, 3.4)	2.9	3 ± 0.2	(2.7, 3.4)
	Oiled	3.0	3.1 ± 0.3	(2.8, 3.7)	3.1	3 ± 0.3	(2.7, 3.5)
	Rehab	3.2	3.3 ± 0.5	(2.6, 4.4)	3.5	3.9 ± 1.4	(3, 7.4)
Chloride (mmol/L)	Control	119	119 ± 2	(114, 121)	117	117 ± 2	(113, 120)
	Oiled	119	120 ± 1	(117, 121)	121	120 ± 2.3	(117, 124)
	Rehab	121	118 ± 6	(104, 123)	117	118 ± 3.6	(114, 124)
TC ionized calcium (mmol/L)	Control	1.3	1.2 ± 0.1	(1.1, 1.4)	1.3	1.2 ± 0.1	(1, 1.4)
	Oiled	1.2	1.2 ± 0.1	(1.1, 1.4)	1.4	1.4 ± 0.1	(1.2, 1.4)
	Rehab	1.2	1.2 ± 0.1	(1, 1.3)	1.3	1.3 ± 0.1	(1, 1.4)
TC anion gap	Control	24	25 ± 24	(20,36)	24	24 ± 2.6	(19, 28)
	Oiled	24	23 ± 2	(20, 25)	23	23 ± 4	(19, 31)
	Rehab	23	23 ± 3	(15, 27)	24	23 ± 2	(20, 26)
Lactate (mmol/L)	Control	5.8	5.5 ± 1.9	(2.1, 9.3)	5.5	5.9 ± 2.3	(2.4, 9.4)
	Oiled	5.4	5.2 ± 1.9	(2.7, 7.3)	5.9	6.1 ± 1.9	(3.3, 9.9)
	Rehab	5.4	5.1 ± 2	(1.7, 2.5)	5.8	5.7 ± 1.5	(3.1, 7.2)
Glucose (mg/dL)	Control	314	318 ± 59	(230, 409)	334	337 ± 45	(251, 393)
	Oiled	310	312 ± 35	(274, 377)	299	297 ± 30	(240, 334)
	Rehab	276	288 ± 47	(232, 383)	293	302 ± 46	(237, 367)
TC pH	Control	7.40	7.4 ± 0.05	(7.3, 7.5)	7.40	7.38 ± 0.04	(7.3, 7.4)
	Oiled	7.35	7.35 ± 0.05	(7.3, 7.4)	7.35	7.35 ± 0.05	(7.3, 7.4)
	Rehab	7.40	7.36 ± 0.05	(7.3, 7.4)	7.40	7.36 ± 0.05	(7.3, 7.4)
TC pCO_2_ (mmHg)	Control	32	30 ± 7	(21, 40)	35	35 ± 3	(32, 41)
	Oiled	35	35 ± 3	(32, 42)	36	36 ± 7	(27, 50)
	Rehab	34	35 ± 6	(46, 19)	37	38 ± 5	(31, 46)
TC pO_2_ (mmHg)	Control	52	53 ± 7	(45, 66)	50	49 ± 4	(43, 57)
	Oiled	50	50 ± 2	(47, 53)	51	50 ± 5	(42, 62)
	Rehab	52	51 ± 6	(42, 60)	51	52 ± 3	(48, 57)
TC bicarbonate (mmol/L)	Control	16	16 ± 3	(11, 22)	18	18 ± 2	(14, 20)
	Oiled	17	17 ± 2	(14, 20)	19	17 ± 3	(12, 21)
	Rehab	17	17 ± 3	(11, 23)	19	18 ± 3	(14, 23)
Total WBC (×10^3^/μL)	Control	10.0	10.1 ± 1.1	(9, 12)	11.0	10.9 ± 1.7	(8, 13)
	Oiled	11.0	10.9 ± 1.1	(9, 13)	10.0	10.1 ± 2	(7, 13)
	Rehab	10.0	9.8 ± 1.3	(7, 11)	9.0	9.2 ± 1.6	(7, 12)
Heterophils (×10^3^/μL)	Control	4.5	4.5 ± 0.6	(3.4, 5.3)	4.9	5.1 ± 1.2	(3.8, 7.2)
	Oiled	5.0	5.1 ± 1	(4, 7)	5.5	5.4 ± 1.9	(2.1, 7.8)
	Rehab	4.5	4.3 ± 1.2	(2.3, 5.5)	4.7	5.1 ± 1.1	(3.6, 7)
Lymphocytes (×10^3^/μL)	Control	2.4	2.3 ± 0.1	(2.2, 2.4)	2.7	2.6 ± 0.4	(1.8, 3.1)
	Oiled	2.5	2.5 ± 0.3	(2.2, 3.1)	2.3	2.3 ± 0.6	(1.4, 3.2)
	Rehab	2.3	2.2 ± 0.4	(1.4, 2.7)	1.9	2.1 ± 0.7	(0.8, 3)
Monocytes (×10^3^/μL)	Control	0.0	0 ± 0.1	(0, 0.1)	0.0	0 ± 0.1	(0, 0.1)
	Oiled	0.1	0.1 ± 0.1	(0, 0.1)	0.0	0 ± 0	(0, 0)
	Rehab	0.0	0 ± 0.1	(0, 0.1)	0.0	0 ± 0	(0, 0.1)
Eosinophils (×10^3^/μL)	Control	0.0	0 ± 0	(0, 0)	0.0	0 ± 0	(0, 0)
	Oiled	0.0	0 ± 0	(0, 0)	0.0	0 ± 0	(0, 0)
	Rehab	0.0	0 ± 0	(0, 0)	0.0	0 ± 0	(0, 0)
Basophils (×10^3^/μL)	Control	0.0	0 ± 0	(0, 0)	0.0	0 ± 0	(0, 0)
	Oiled	0.0	0 ± 0	(0, 0)	0.0	0 ± 0	(0, 0)
	Rehab	0.0	0 ± 0	(0, 0)	0.0	0 ± 0	(0, 0)
Reticulocytes (%)	Control	0.7	0.8 ± 0.3	(0.5, 1.6)	0.7	0.6 ± 0.2	(0.3, 0.9)
	Oiled	1.6	1.5 ± 0.5	(0.7, 2.1)	1.1	1.3 ± 0.7	(0.4, 3)
	Rehab	1.0	1 ± 0.4	(0.3, 1.7)	0.6	0.8 ± 0.4	(0.3, 1.4)
		**22 DPO**			**31 DPO**		
**Analytes**	**Group**	**Median**	**Mean** **±** **SD**	**(Min, Max)**	**Median**	**Mean** **±** **SD**	**(Min, Max)**
Weight (g)	Control	440	473 ± 55	(414, 552)	428	458 ± 54	(396, 546)
	Oiled	430	437 ± 41	(396, 500)	444	468 ± 57	(410, 576)
	Rehab	488	477 ± 43	(412, 556)	472	472 ± 45	(408, 544)
Body temperature (°C)	Control	42.8	42.7 ± 0.4	(42.1, 43.2)	42.2	42.1 ± 0.8	(40.5, 43.4)
	Oiled	43.4	43.1 ± 1	(40.3, 43.9)	42.6	42.8 ± 0.4	(42.3, 43.5)
	Rehab	43.0	43 ± 0.3	(43, 44)	42.1	42.1 ± 0.6	(40.7, 42.7)
PCV (spun) (%)	Control	48	48 ± 4	(42, 53)	53	52 ± 3	(46, 55)
	Oiled	47	43 ± 11	(12, 49)	46	46 ± 2	(43, 49)
	Rehab	47	46 ± 3	(40, 49)	50	49 ± 3	(43, 44)
Hematocrit (EPOC) (%)	Control	41	41 ± 2	(36, 43)	43	43 ± 2	(40, 46)
	Oiled	38	35 ± 9	(9, 39)	36	36 ± 2	(34, 38)
	Rehab	38	37 ± 2	(34, 39)	39	39 ± 3	(33, 44)
Total Solids (g/dL)	Control	4.2	4.2 ± 0.8	(3, 5.8)	4.9	4.6 ± 0.5	(3.8, 5.2)
	Oiled	4.4	4.1 ± 1	(1.4, 5)	4.3	4.5 ± 0.4	(4, 5.1)
	Rehab	4.2	4.3 ± 0.8	(3.5, 6)	4.2	4.2 ± 0.3	(3.6, 4.6)
Sodium (mmol/L)	Control	156	156 ± 2	(153, 158)	155	155 ± 1	(153, 157)
	Oiled	157	157 ± 2	(153, 160)	155	156 ± 2	(153, 159)
	Rehab	156	156 ± 2	(153, 159)	158	157 ± 2	(153, 159)
Potassium (mmol/L)	Control	3.2	3.2 ± 0.1	(2.9, 3.3)	3.2	3.1 ± 0.2	(2.8, 3.6)
	Oiled	3.3	3.4 ± 0.4	(2.9, 4.1)	3.0	3 ± 0.4	(2.1, 3.5)
	Rehab	3.4	3.4 ± 0.4	(2.9, 4.1)	3.0	3 ± 0.4	(2.3, 3.4)
Chloride (mmol/L)	Control	118	118 ± 3	(115, 124)	120	119 ± 3	(115, 122)
	Oiled	120	120 ± 2	(118, 123)	120	120 ± 3	(116, 124)
	Rehab	117	118 ± 3	(113, 121)	120	119 ± 3.2	(113, 123)
TC ionized calcium (mmol/L)	Control	1.2	1.2 ± 0.1	(1.1, 1.3)	1.2	1.2 ± 0.1	(1.1, 1.3)
	Oiled	1.3	1.3 ± 0.1	(1.1, 1.4)	1.2	1.2 ± 0.1	(1.1, 1.3)
	Rehab	1.3	1.3 ± 0.1	(1.2, 1.4)	1.2	1.2 ± 0.1	(0.9, 1.3)
TC anion gap	Control	23	23 ± 2.4	(19, 27)	21	22 ± 3	(19, 27)
	Oiled	23	24 ± 2	(22, 27)	22	22 ± 2	(19, 27)
	Rehab	24	24 ± 2	(21, 27)	23	22 ± 3	(17, 25)
Lactate (mmol/L)	Control	4.5	5.1 ± 1.6	(3.3, 8.1)	4.8	5.7 ± 2.2	(3.2, 9.6)
	Oiled	5.3	5.7 ± 2.3	(2.8, 10.7)	5.9	6.1 ± 1.4	(3.7, 8.2)
	Rehab	5.6	5.4 ± 1.9	(1.7, 7.8)	6.2	5.8 ± 1.4	(3.6, 7.6)
Glucose (mg/dL)	Control	326	314 ± 65	(214, 409)	325	346 ± 41	(297, 425)
	Oiled	251	264 ± 33	(221, 321)	337	343 ± 56	(238, 419)
	Rehab	302	293 ± 48	(235, 353)	348	341 ± 30	(296, 371)
TC pH	Control	7.40	7.39 ± 0.03	(7.3, 7.4)	7.40	7.39 ± 0.06	(7.3, 7.5)
	Oiled	7.35	7.36 ± 0.07	(7.3, 7.5)	7.30	7.32 ± 0.04	(7.3, 7.4)
	Rehab	7.30	7.34 ± 0.05	(7.3, 7.4)	7.30	7.35 ± 0.08	(7.3, 7.5)
TC pCO_2_ (mmHg)	Control	33	34 ± 3	(30, 38)	34	33 ± 3	(26, 37)
	Oiled	38	36 ± 7	(20, 45)	36	36 ± 3	(33, 40)
	Rehab	35	38 ± 5	(32, 47)	37	38 ± 7	(26, 48)
TC pO_2_ (mmHg)	Control	52	51 ± 4	(45, 56)	52	52 ± 3	(47, 58)
	Oiled	51	51 ± 5	(46, 58)	49	50 ± 6	(45, 63)
	Rehab	49	51 ± 4	(48, 60)	49	49 ± 3	(45, 55)
TC bicarbonate (mmol/L)	Control	17	18 ± 2	(15,20)	17	17 ± 1	(15,19)
	Oiled	16	17 ± 3	(12, 22)	15	16 ± 2	(14, 20)
	Rehab	18	17 ± 2	(15, 20)	18	18 ± 2	(16, 20)
Total WBC (×10^3^/μL)	Control	11.0	11.2 ± 1.1	(10,13)	10.5	10 ± 2.2	(7, 13)
	Oiled	11.0	10.6 ± 1.7	(8, 13)	11.0	10.4 ± 2.5	(7, 13)
	Rehab	12.0	11.1 ± 2	(7, 13)	10.0	10 ± 2.4	(7, 13)
Heterophils (×10^3^/μL)	Control	5.0	5 ± 1.5	(2.8, 7)	3.7	4.3 ± 1.9	(1.9, 7.8)
	Oiled	5.0	5.4 ± 1.8	(2.7, 8)	5.6	5.1 ± 1.3	(2.9, 6.8)
	Rehab	6.6	6.2 ± 2.1	(3.2, 9.1)	5.0	5.2 ± 1.7	(3.2, 9)
Lymphocytes (×10^3^/μL)	Control	2.5	2.5 ± 0.4	(2, 3.3)	2.2	2.2 ± 0.5	(1.4, 3.1)
	Oiled	2.4	2.4 ± 0.4	(1.9, 3)	2.7	2.5 ± 0.6	(1.7, 3.3)
	Rehab	2.5	2.4 ± 0.4	(1.7, 2.8)	2.2	2.3 ± 0.5	(1.7, 3.1)
Monocytes (×10^3^/μL)	Control	0.0	0 ± 0	(0, 0.1)	0.0	0 ± 0	(0, 0.1)
	Oiled	0.0	0 ± 0	(0, 0.1)	0.0	0 ± 0	(0, 0)
	Rehab	0.0	0 ± 0	(0, 0)	0.0	0 ± 0	(0, 0.1)
Eosinophils (×10^3^/μL)	Control	0.0	0 ± 0	(0, 0)	0.0	0 ± 0	(0, 0)
	Oiled	0.0	0 ± 0	(0, 0)	0.0	0 ± 0	(0, 0)
	Rehab	0.0	0 ± 0	(0, 0)	0.0	0 ± 0	(0, 0)
Basophils (×10^3^/μL)	Control	0.0	0 ± 0	(0, 0)	0.0	0 ± 0	(0, 0)
	Oiled	0.0	0 ± 0	(0, 0)	0.0	0 ± 0	(0, 0)
	Rehab	0.0	0 ± 0	(0, 0)	0.0	0 ± 0	(0, 0)
Reticulocytes (%)	Control	1.1	1.2 ± 0.4	(0.7, 2)	0.9	1.1 ± 0.8	(0.3, 2.7)
	Oiled	1.6	1.5 ± 0.6	(0.4, 2.3)	1.5	1.5 ± 0.5	(0.7, 2.6)
	Rehab	0.8	1.1 ± 0.6	(0.4, 2)	0.9	1 ± 0.6	(0.4, 2.2)

*TC, temperature corrected; SD, standard deviation*.

Estimated reticulocytes significantly differed among treatment groups but did not change over time nor exhibit an interaction between group and time (Table 2). Non-rehabilitated-oiled RBGUs had a 1.3 ± 0.1% higher estimated reticulocyte percentage than both rehabilitated-oiled and control RBGUs. Body weight, body temperature, total solids, sodium, potassium, chloride, TC ionized calcium, lactate, glucose, TC anion gap, TC pH, TC pO_2_, TC bicarbonate, total WBC, heterophils, and lymphocytes did not differ among treatment groups, change over time, or exhibit an interaction between group and time.

**Figure 1:**
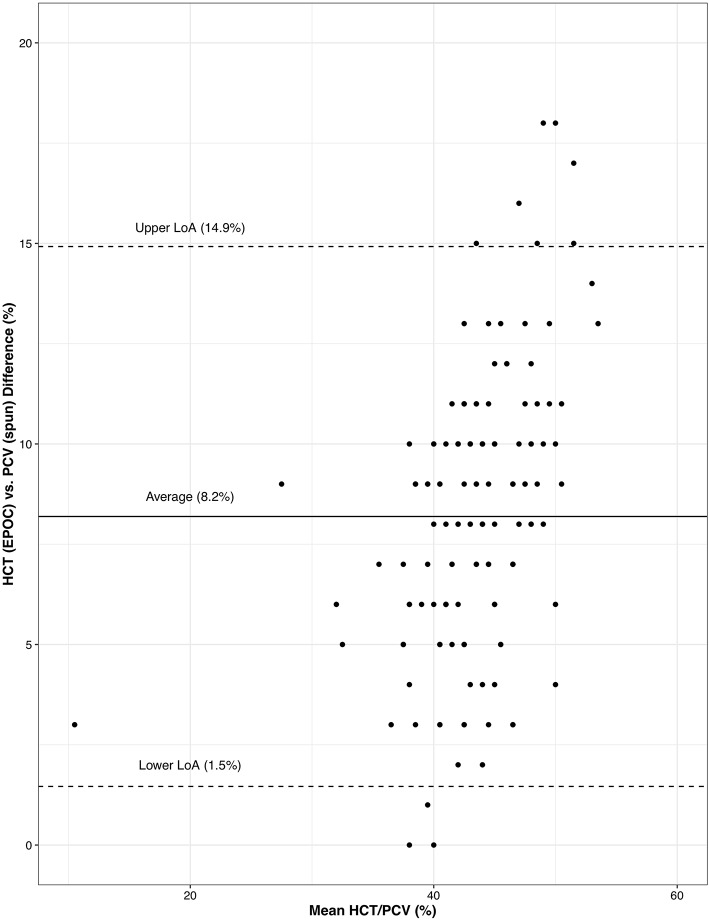
A Bland-Altman plot of the diagnostic difference between hematocrit as measured by the Element POC Blood Gas and Electrolyte Analyzer—HCT (EPOC)—and PCV determined manually following high-speed centrifugation of microhematocrit tubes—PCV (spun). Values on the x-axis represent the mean HCT/PCV between the two methods for a given Ring-billed Gull (*Larus delawarensis*) and values on the y-axis represent the difference between the two methods for the same Ring-billed Gull. Levels of agreement (LoA) represent a 95% confidence interval around the average difference.

HCT (EPOC) and PCV (spun) were significantly different [*t*_(145)_ = 28.78, *p* < 0.001]—the HCT (EPOC) was on average 8.2% (95% CI: 1.5–14.9%) less than PCV (spun). The concordance correlation coefficient between HCT (EPOC) and PCV (spun) was 0.35 (95% CI: 0.28–0.41) suggesting low concordance in their results. A Bland-Altman plot of the relationship between HCT (EPOC) and PCV (spun) shows considerable variability in the discrepancy between the two methods, but no obvious trend (Figure 1).

## Discussion

Brought to near extinction due to anthropogenic forces in the late nineteenth century, RBGUs have rebounded and become one of the most common and widespread gull species in North America. We are the first to report blood data reference intervals for wild RBGUs, a potential resource for future comparative studies or clinical work. RBGU PCV (spun) was similar to PCV data previously reported for other gull species (35–38). Total solids were lower than means reported in Kelp Gulls (*Larus dominicanus*) (37, 38), but birds sampled in both of these studies foraged on high-protein discards from the fishing industry which likely resulted in higher plasma protein concentrations. Glucose concentrations were within the range observed in Kelp Gulls and other seabirds (35, 37, 38). On average, RBGUs had a higher lymphocytes compared to heterophils, a finding seen in Great Black-backed Gulls (*L. marinus*) and Herring Gulls (*L. argentatus*) (39), but opposite of a pattern seen in Kelp Gulls (37).

Our results support our hypothesis that RBGUs develop some degree of anemia secondary to external oil exposure, similar to other avian species. The anemia seen in oiled RBGUs falls within the range of anemia found in externally oiled birds sampled shortly after the Deepwater Horizon oil spill which had on average a 4–19% lower PCV than non-oiled birds (40). Oiled RBGUs may have had a milder anemia compared to some of the birds sampled after Deepwater Horizon due to their easy access to nutritious, oil-free food, and limited need to expend energy foraging. Although control RBGUs had higher HCT (EPOC) than both non-rehabilitated and rehabilitated-oiled RBGUs over the study period, all RBGUs exhibited anemia after 0 DPO suggesting that both external oiling and aspects of captivity or repeated handling cause persistent mild anemia. The implication that captivity can induce anemia in wild birds over time supports previous experimental work; both orally exposed to oil and non-exposed Rhinoceros Auklets (*Cerorhinca monocerata*) had on average a 10% lower PCV after 3 weeks in captivity (41). Given our RBGUs were group housed in large outdoor flight pens that encouraged natural behaviors, the development of anemia secondary to captivity, regardless of oil exposure, is potentially due to alterations in physiological demands or the stress of repeated handling, rather than the sedentary nature of captivity.

While non-rehabilitated-oiled RBGUs had a higher estimated reticulocyte percentage than both rehabilitated-oiled RBGUs and control RBGUs, all three treatment groups failed to show increased RBC regeneration in the face of a persistent mild anemia, given reticulocyte percentages vary between 1 and 5% in healthy birds (27). This finding contrasts our hypothesis that rehabilitated birds would return to pre-exposure values quicker than non-rehabilitated birds as well as field observations made after the Deepwater Horizon oil spill that found externally oiled birds had on average 27-40% higher reticulocyte percentages (40). However, the absence of a regenerative response is similar to results from previous experimental studies. Rhinoceros Auklets orally exposed to oil as well as Double-crested Cormorants (*Phalacocorax auritis*) orally and externally exposed to oil showed regenerative, but inadequately compensated, responses to anemia with PCV never returning to levels recorded post-capture and pre-oiling (41, 42). The lack of compensatory RBC regeneration despite persistent anemia suggests a lack of production in hematopoietic tissue which may be caused by oil-induced bone marrow suppression or potential changes in physiological demands wild birds experience in captivity resulting in a “resetting” of homeostatic RBC levels.

Although sublethal oil exposure has long been suspected of causing immunosuppression in birds, the effects of oil toxicosis in rehabilitated or experimental birds is confounded with captivity-related stress. RBGUs showed low heterophil to lymphocyte ratios on 0 DPO, suggesting the birds were well-acclimated to captivity prior to external oiling given heterophils increase and lymphocytes decrease in response to stress (43). Following oil exposure, no difference in total WBC, heterophil, lymphocyte, monocyte, eosinophil, and basophil counts were found among treatment groups. This is inconsistent with our hypothesis and implies that moderate external oiling does not alter circulating WBCs. Laughing Gulls (*Leucophaeus atricilla*) offered fish injected with oil also showed no changes in circulating WBCs (44), whereas Common Terns (*Sterna hirundo*), sampled 14–21 days after the Buzzards Bay oil spill, exhibited severely depressed total WBC and lymphocyte counts as well as markedly elevated heterophil counts and heterophil/lymphocyte ratios (15). While further research is warranted, the acute stress of capturing and handling wild birds is potentially a factor in explaining the discrepancy between experimental studies showing no oil-induced immunosuppression and field observations showing some degree of immunosuppression. It is also possible that immunosuppression in experimentally oiled birds is mitigated by the adequate husbandry and nutrition provided during experimental studies. The WBC estimate methodology used herein was developed from unpublished studies and has not been validated or verified by independent review. Therefore, comparisons of our WBC data to other studies needs to be done cautiously.

The significant interaction between non-rehabilitated-oiled RBGUs' HCT (EPOC) suggests that chronic sublethal external oil exposure may result in ongoing anemia. While the control and rehabilitated-oiled RBGUs' HCT (EPOC) gradually increased toward baseline levels between 15 and 31 DPO, the non-rehabilitated-oiled RBGUs' HCT (EPOC) continued to decrease. The diagnostic disagreement between the EPOC and PCV determined manually is not surprising given the difference in methodologies. Veterinary point-of-care analyzers measure hematocrit via whole blood conductometry in which a higher proportion of RBCs decreases electrical conductivity. Consequently, many veterinary point-of-care analyzers likely underestimate avian hematocrit due to the difference in conductivity between avian nucleated RBCs and mammalian non-nucleated RBCs. Measurements of hematocrit performed by the VetScan i-STAT 1 Analyzer (Abaxis Inc., Union City, California) have been previously shown to be on average 15% lower than PCV determined manually in Bar-headed Geese (*Anser indicus*) (45) suggesting that the magnitude of underestimating hematocrit may vary amongst species or veterinary point-of-care analyzers. In addition to our limited sample size, possible limitations of our study include significant differences in weather across the study period, Element POC cartridge temperature, and/or observer effects, given sampling was conducted outside and multiple observers performed WBC differentials. Other limitations of our study include the RBGUs' aforementioned easy access to nutritious, oil-free food and limited need to expend energy foraging as well as the availability of heat lamps minimizing the adverse effects of oil on thermoregulation. Despite accounting for the heterogeneity of individual RBGUs in our LMMs as a random effect, the small sample size per treatment group could explain the significant interaction found with HCT (EPOC) but not PCV (spun) or the lack of differences among treatment groups for some analytes.

## Conclusions

Our study is the first to report hematological, biochemical, and blood gas analytes in birds following sublethal oil exposure and rehabilitation. We found that both sublethal oil exposure and aspects of captivity caused a mild non-regenerative anemia, suggesting the etiology of anemia seen in oiled birds undergoing rehabilitation is possibly multifactorial. Given that moderately oiled gulls in our study had subtle, but potentially not insignificant clinicopathological abnormalities, gulls affected by sublethal external oiling are likely strong candidates for rescue and rehabilitation. Furthermore, oiled gulls did not develop any clinicopathological derangements post-rehabilitation, suggesting current standard practices for rehabilitation cause minimal morbidity in stable, moderately oiled gulls.

## Data Availability Statement

The datasets generated for this study are available on request to the corresponding author.

## Ethics Statement

This animal study was reviewed and approved by the USDA/APHIS/WS National Wildlife Research Center Institutional Animal Care and Use Committee.

## Author Contributions

ND, KH, and SS developed this study's design and oversaw all aspects of the project. JE lead efforts to capture study animals and assisted with their routine husbandry. LW provided the veterinary point of care analyzer used in this study as well as veterinary expertise. ND was responsible for statistical analysis and primarily writing this manuscript. All authors assisted with data collection and played a significant role in editing/reviewing this manuscript.

### Conflict of Interest

The authors declare that the research was conducted in the absence of any commercial or financial relationships that could be construed as a potential conflict of interest.
